# EGFR-selective activation of CD27 co-stimulatory signaling by a bispecific antibody enhances anti-tumor activity of T cells

**DOI:** 10.3389/fimmu.2023.1191866

**Published:** 2023-07-20

**Authors:** Vinicio Melo, Levi Collin Nelemans, Martijn Vlaming, Harm Jan Lourens, Valerie R. Wiersma, Vrouyr Bilemjian, Gerwin Huls, Marco de Bruyn, Edwin Bremer

**Affiliations:** ^1^ Department of Hematology, University Medical Center Groningen, University of Groningen, Groningen, Netherlands; ^2^ Department of Obstetrics & Gynecology, University Medical Center Groningen, University of Groningen, Groningen, Netherlands

**Keywords:** immunotherapy, bispecific antibody, CD27, EGFR, T cell, co-stimulation

## Abstract

A higher density of tumor infiltrating lymphocytes (TILs) in the tumor microenvironment, particularly cytotoxic CD8^+^ T cells, is associated with improved clinical outcome in various cancers. However, local inhibitory factors can suppress T cell activity and hinder anti-tumor immunity. Notably, TILs from various cancer types express the co-stimulatory Tumor Necrosis Factor receptor CD27, making it a potential target for co-stimulation and re-activation of tumor-infiltrated and tumor-reactive T cells. Anti-cancer therapeutics based on exploiting CD27-mediated T cell co-stimulation have proven safe, but clinical responses remain limited. This is likely because current monoclonal antibodies fail to effectively activate CD27 signaling, as this receptor requires higher-order receptor cross-linking. Here, we report on a bispecific antibody, CD27xEGFR, that targets both CD27 and the tumor antigen, epidermal growth factor receptor (EGFR). By targeting EGFR, which is commonly expressed on carcinomas, CD27xEGFR induced cancer cell-localized crosslinking and activation of CD27. The design of CD27xEGFR includes an Fc-silent domain, which is designed to minimize potential toxicity by reducing Fc gamma receptor-mediated binding and activation of immune cells. CD27xEGFR bound to both of its targets simultaneously and triggered EGFR-restricted co-stimulation of T cells as measured by T cell proliferation, T cell activation markers, cytotoxicity and IFN-γ release. Further, CD27xEGFR augmented T cell cytotoxicity in a panel of artificial antigen-presenting carcinoma cell line models, leading to Effector-to-Target ratio-dependent elimination of cancer cells. Taken together, we present the *in vitro* characterization of a novel bispecific antibody that re-activates T cell immunity in EGFR-expressing cancers through targeted co-stimulation of CD27.

## 1 Introduction

The re-activation of tumor-reactive T cells with so-called immune checkpoint inhibitors (ICIs) has translated into remarkable clinical breakthroughs. Specifically, antibodies directed against CTLA-4 and PD-L1/PD-1 have improved therapeutic outcomes, including complete remissions in many solid as well as hematological cancers (as reviewed in (1–3)). ICIs prevent negative feedback on tumor-reactive T cells and re-enable the eradication of cancer cells upon binding of the T cell receptor (TCR) complex to tumor-specific peptides presented in the major histocompatibility complex (MHC). However, not all patients or cancer types respond to current ICI therapies (as reviewed in (4–6)).

One possible explanation for the limited activity of ICI therapy in certain patients and cancer types may be the absence of additional co-stimulatory signals that stimulate tumor-reactive T cells in the tumor microenvironment (TME) (7–9). For example, the inhibition of the co-stimulatory CD40-CD40L axis diminished the effects of PD-L1 checkpoint treatment on exhausted CD8^+^ T cells (10). Moreover, the lack of CD28 co-stimulation has been postulated to be a strong determinant of PD-1 blockade resistance (as reviewed in (11)). In order to provide sufficient co-stimulation, so-called immune co-stimulators (ICS) that target and activate prominent co-stimulatory receptors (e.g., CD28, CD40, 4-1BB, CD27, and OX40) have been developed and are currently undergoing clinical evaluation (12–18).

A prominent co-stimulatory receptor family involved in T cell activation is the Tumor Necrosis Factor Receptor Super Family (TNFRSF). Within this superfamily, CD27 (TNFRSF7) has emerged as a potential target for co-stimulatory therapy, yielding clinical benefits in a select group of hematological and solid tumors (19–22). CD27 is not only constitutively expressed on the majority of both CD4^+^ and CD8^+^ T cells, but is also highly expressed on the majority of tumor infiltrating lymphocytes (TILs). Therefore, the activation of CD27 signaling is regarded as a potentially effective therapeutic anti-cancer strategy (23–27).

When activated by its ligand CD70, CD27 promotes the proliferation of T cells and their differentiation into effector and memory T cells (28–31). Importantly, CD27 co-stimulatory signaling is only efficiently activated upon the simultaneous occurrence of two events: (1) TCR-mediated recognition of and binding to tumor-specific peptides presented in the MHC of antigen presenting cells; and (2) crosslinking of CD27 (13, 32, 33). For instance, treatment with the CD27 agonistic antibody Varlilumab upregulated cytokine secretion upon continuous TCR-triggering, whereas pre-activated T cells without continuous TCR-triggering did not respond to Varlilumab (22, 33). Consequently, immunotherapies targeting CD27 have resulted in safer therapeutic outcomes than co-stimulatory approaches that can also operate TCR-independently, such as CD28 co-stimulation (34, 35). However, treatment with Varlilumab yielded only one (1/10) complete response and one stable disease (SD) in Hodgkin lymphoma and three (3/18) SD in B cell non-Hodgkin lymphoma (21). Further, in a trial with 31 patients with advanced solid tumors, Varlilumab yielded one partial response, with eight patients experiencing SD (22). Thus, the therapeutic effect of single CD27 targeting with Varlilumab in patients is limited.

The disappointing clinical results with Varlilumab may be attributable to suboptimal receptor crosslinking, as effective CD27 downstream signaling requires a hexameric ligand format and a hexameric CD27 complex (32, 36). Furthermore, CD27 receptor hexamerization and agonism is dependent on targeting specific extracellular CD27 epitopes and the application of Fc-engineering strategies that amplify affinity to Fc gamma receptors (FcγRs) (37). Current CD27-agonistic monoclonal antibodies (mAbs) do not efficiently promote CD27 hexamerization as single agents and require a scaffold, such as Fc receptor (FcR)-expressing immune cells. For instance, MK-5890, a novel CD27 agonistic antibody, showed increased agonistic activity with the occurrence of Fc-FcγR interactions (38). A potential approach to overcome this limitation is selective receptor crosslinking using a bispecific antibody (bsAb). Binding of a bsAb to a tumor-associated antigen can serve as a cross-linking platform for CD27 on T cells (39). Indeed, in preclinical studies, CDX-527, a tetravalent PD-L1 and CD27-targeting bsAb, induced CD27-mediated T cell co-stimulation through cross-linking by PD-L1 more effectively than the parental antibodies combined (40).

In the current study, we aimed to evaluate whether CD27 co-stimulation could be restricted to epidermal growth factor receptor (EGFR)-positive cancer (EGFR^+^). While EGFR is ubiquitously expressed, EGFR expression is upregulated in several carcinomas and associates with tumor progression and angiogenesis (as reviewed in (41–43)). Hereto, we created an ICS-bsAb in a Dual-Variable Domain Immunoglobulin (DVD-Ig) format (44) consisting of two antigen-binding fragments (scFv1F5 and scFv425) targeting CD27 and EGFR, respectively. This antibody, termed CD27xEGFR, features an Fc-domain with LALAPG point mutations to yield an Fc-silent human IgG1, reducing FcR mediated antibody effector functions. In this format, the bsAb CD27xEGFR is designed to be minimally active ‘en route’. Once CD27xEGFR binds to EGFR^+^ cancer cells, the CD27-targeting domain can provide multivalent and tumor-localized crosslinking of CD27, potentially reducing off-tumor side effects. Besides serving as a cross-linking platform to facilitate CD27 co-stimulatory signaling, the EGFR targeting moiety of CD27xEGFR may also contribute through direct EGFR blocking, which is already a well-established therapeutic strategy for several epithelial tumors (45, 46). Hence, CD27xEGFR is designed to re-activate anti-tumor immunity safely and effectively in EGFR^+^ cancer cells.

## 2 Materials and methods

### 2.1 The Cancer Genome Atlas dataset analysis

A PanImmune Feature Matrix of Immune Characteristics as described in (47) was used to obtain lymphocytic infiltrate signature scores (based on the following 18 markers defined in (48): CCL5, CD19, CD37, CD3D, CD3E, CD3G, CD3Z, CD79A, CD79B, CD8A, CD8B1, IGHG3, IGJ, IGLC1, CD14, LCK, LTB, MS4A1) across multiple tumor-samples, containing The Cancer Genome Atlas (TCGA) Participant Barcodes. Batch effect normalized TCGA PAN CANCER CD27 and EGFR expression levels were obtained *via* Xena Hub (49), which consisted of TCGA data from broad GDAC firehose, that was normalized by RSEM (RNA-seq by Expectation-Maximization) and batch corrected *via* EB++ (Empirical Bayes++) (synapse ID: syn4976363). Expression levels were matched with lymphocytic infiltrate signature scores based on TCGA Participant Barcodes, and linear regressions were performed in GraphPad Prism 8.0.2. To visualize EGFR expression across multiple tumor-types, violin plots were generated.

### 2.2 Single cell mRNA sequencing data analysis

The dataset from the Tumor Immune Cell Atlas study (50) consisting of 13 different cancer types, 217 patients, and 526,261 cells was downloaded in the form of a RDS file containing the Seurat object. The data was ingested into Seurat V4 in R language version 4.0.3. The integrated single-cell RNA sequencing (scRNA-seq) data sets were collected as described before by Nieto et al. (50). In brief, after integration, the cells were divided into 25 clusters representing major immune cell types including 12 T cell types. To verify the robustness of the clusters and the associated signatures, a random forest classifier was used to assign cell annotations. A fivefold cross-validation was performed to assess biases and variance (50). The following T cell types were included in our study: Regulatory T cells, T helper cells, Th17 cells, recently activated CD4^+^ T cells, Naïve-memory CD4^+^ T cells, Transitional memory CD4^+^ T cells, Naïve T cells, Proliferative T cells, Pre-exhausted CD8^+^ T cells, Cytotoxic CD8^+^ T cells, Effector memory CD8^+^ T cells, and Terminally exhausted CD8^+^ T cells (For the key markers per subtype, see **Supplementary Table 1**). Differential expression was calculated by using the FindMarkers function from Seurat with MAST as the method of choice (51).

### 2.3 Antibodies

Polyclonal antibody (pAb) Goat anti-human Ig-PE (cat# 2040-09, Southern Biotech, Birmingham, AL, USA), monoclonal antibodies (mAb): anti-CD27-APC (cat# 302810, clone O323, BioLegend, San Diego, CA, USA) (also used as CD27 mAb), anti-EGFR-FITC (cat# sc-120 FITC, clone 528, Santa Cruz Biotechnology, Dallas, TX, USA), anti-CD25-APC (cat# 302610, clone BC96, BioLegend), anti-CD4-FITC (cat# 300506, clone RPA-T4, BioLegend), anti-CD8-Brilliant Violet 421 (cat# 344748, clone SK1, BioLegend), mouse (IgG2A) (mAb 425) (Cat# EWI020, Kerafast, Boston, MA, USA), anti-Myc mAb Alexa Fluor 647 (cat# 2233, clone 9B11, Cell Signaling, Danvers, MA, USA). Atezolizumab was obtained from the pharmacy of the UMCG (Groningen, the Netherlands).

### 2.4 Cell lines and transfectants

The following wild type (WT) cell lines were obtained from the American Type Culture Collection (ATCC): A431, MDA-MB-231, ES-2, DLD-1, FaDu, and OVCAR-3. OVCAR-3.EGFR knock-out (KO) cells were a kind gift from prof. dr. Helfrich (UMCG/Dept of Surgery, Groningen, the Netherlands). HT1080.CD27 is previously described in (39) and is a kind gift from prof. dr. Harald Wajant (University of Wuerzburg, Wuerzburg, Germany). An overview of the cell lines, including tissue, cell type, cancer type, species, source and transduced genes, can be found in **Table 1**. Cells were cultured in RPMI-1640 (cat# 21875034, Gibco, Thermo Fisher Scientific, Waltham, MA, USA) or DMEM (cat# 11965092, Gibco, Thermo Fisher Scientific), supplemented with 10% fetal calf serum (FCS)(cat# F7524, Thermo Fisher Scientific) at 37°C/5% CO_2_. The artificial scFvCD3 (UchtV1 anti-CD3 antibody fragment)-presenting cell lines MDA-MB-231^scFvCD3^, ES-2^scFvCD3^, DLD-1^scFvCD3^, FaDu^scFvCD3^, OVCAR-3^scFvCD3^, and OVCAR-3^scFvCD3^EGFR^KO^ are based on the lentiviral synNotch receptor construct pHR_PGK_antiCD19_ synNotch_Gal4VP64, which was a gift from Wendell Lim (Addgene plasmid #79125; http://n2t.net/addgene:79125; RRID : Addgene_79125) (52). The anti-CD19 scFv was replaced with the scFvCD3 UCHT-1v9 using Gibson cloning (cat# E5510S, New England BioLabs, Ipswich, MA, USA), yielding pHR_PGK_scFvCD3_synNotch_Gal4VP64. Lentivirus was produced by transient transfection of HEK293T cells with transfer vector, psPAX2, and pCMV-VSV-G packaging system using FuGENE (cat# E2312, Promega, Madison, WI, USA) according to manufacturer’s recommendations. Viral supernatant was collected and filtered through a 0.45 μm filter (cat# SLHVR13SL, Millipore, Burlington, MA, USA). Transduction was performed by adding 1.5 mL viral supernatant to 1.5 mL of RPMI containing 2.5 × 10^5^ pre-seeded cells in a 6 well tissue culture plate (cat# 3516, Corning Inc., Corning, NY, USA) in the presence of 4 μg/mL polybrene (cat# TR-1003, Sigma-Aldrich, Saint Louis, MO, USA). Transduced cells were sorted for expression of a Myc-tag (present at the N-terminus of the CD3 scFv) with a cell sorter model SH-800s (Sony Biotechnology, San Jose, CA, USA). Before each experiment, ES-2^scFvCD3^, DLD-1^scFvCD3^, FaDu^scFvCD3^, OVCAR-3^scFvCD3^ and OVCAR-3 ^scFvCD3^.EGFR^KO^ cells stably expressing scFvCD3 were characterized for their expression of EGFR, scFvCD3 and CD27, maintaining a consistent fold change from the isotype (**Supplementary Figure 3**). Cells were also transduced with lentivirus containing vector pLKO.1 mCherry, which was a gift from Oskar Laur (Addgene plasmid #128073; http://n2t.net/addgene:128073; RRID : Addgene_128073), producing the corresponding mCherry-expressing cells lines for visualization in the cytotoxicity assays.

**Table 1:** Characteristics of cell lines employed in this study.

Cell line	Tissue	Cell type	Cancer type	Species	Source	Transduced genes
A431	Skin	Epithelial	Epidermoid Carcinoma	Human	ATCC	
HT1080	Connective tissue	Epithelial	Fibrosarcoma	Human	prof. dr. Harald Wajant*	CD27
MDA-MB-231	Breast	Epithelial	Adenocarcinoma	Human	ATCC	scFvCD3 synNotch
ES-2	Ovary	Fibroblast	Clear cell Carcinoma	Human	ATCC	scFvCD3 synNotch, mCherry
DLD-1	Large intestine; Colon	Epithelial	Adenocarcinoma	Human	ATCC	scFvCD3 synNotch, mCherry
FaDu	Pharynx	Epithelial	Squamous Cell Carcinoma	Human	ATCC	scFvCD3 synNotch, mCherry
OVCAR-3	Ovary	Epithelial	Adenocarcinoma	Human	ATCC	scFvCD3 synNotch, mCherry
OVCAR-3.EGFR^KO^	Ovary	Epithelial	Adenocarcinoma	Human	prof. dr. Helfrich**	scFvCD3 synNotch, mCherry

*Provided by Prof. Dr. Harald Wajant, University of Wuerzburg, Wuerzburg, Germany.

**Provided by Prof. Dr. Helfrich, UMCG/Dept of Surgery, Groningen, the Netherlands.

Further details can be found in Materials and Methods (Section 2.4). ATCC, American Type Culture Collection.

### 2.5 Construction of CD27xEGFR

The bsAb CD27xEGFR was constructed in an scFv-scFv-IgG1 format, containing the antigen-binding fragments scFv1F5, targeting CD27, and scFv425, targeting EGFR. These two scFvs were connected by a flexible glycine-serine (GS) linker, consisting of a (GGGS)_3_ sequence. The Fc domain of the antibody was designed with LALAPG mutations (L234A, L235A, and P329G) (53) in order to create an effector silent IgG molecule. Another GS linker, with the same (GGGGS)_3_ sequence, connects the scFv EGFR to the IgG1 Fc domain. The antibody was produced by Evitria (Schlieren, Switzerland). Supernatant was harvested by centrifugation, filtered (0.2 μm filter), whereupon antibody was purified using MabSelect SuRe (cat# GE17-5438-01, Merck KGaA, Darmstadt, Germany). Purity was evaluated by analytical size exclusion chromatography with an AdvanceBio SEC column (300A 2.7 um 7.8 x 300 mm) (cat# PL1180-5301, Agilent, Santa Clara, CA, USA) and Dulbecco’s phosphate-buffered saline (DPBS) (cat# 14190144, Gibco, Thermo Fisher Scientific) as running buffer at 0.8 mL/min. CD27xEGFR was successfully purified up to 98.6% with only minor amounts of degradation product (**Supplementary Figure 1A**). Endotoxin content was measured with the Charles River Endosafe PTS system (cat# PTS150K, Wilmington, MA, USA) and was < 1 EU/mg.

### 2.6 Biolayer interferometry assay

Binding of His-CD27 or His-EGFR to CD27xEGFR was analyzed using the BLItz system from ForteBio (cat# 45-5000, ForteBio, Menlo Park, CA, USA). His-CD27 (cat# 10039-H08B1, SinoBiological, Beijing, China), produced using the Baculovirus-Insect Cell expression system, encodes for the extracellular domain of human CD27 (Met1-Ile192) and included a C-terminal polyhistidine (His) tag. His-EGFR (cat# Z03194, GenScript, Rijswijk, the Netherlands), generated with the Sf9 insect cell expression system, encodes for the extracellular domain of human EGFR (Leu25-Ser645), and also featured a C-terminal His tag.

Octet protein A biosensors (cat# 18-5010, Satorius, Göttingen, Germany) were wetted for at least 10 min before use in 100 mM Tris-HCl pH 8 and all samples were diluted in the same buffer. In short, a baseline was run for 30 sec, followed by loading of 8 µg/mL of CD27xEGFR for 120 sec, baseline for 30 sec, association of either 125 nM His-EGFR and/or 500 nM His-CD27 for 120 sec, and dissociation for 120 sec. Atezolizumab (50 µg/mL) was used as a control.

The same protocol was used for binding of CD27xEGFR to immobilized His-CD27 and His-EGFR, with the following exceptions: The use of Octet HIS1K biosensors (cat# 18-5120, Sartorius), loading of 500 nM His-EGFR or His-CD27 and association of CD27xEGFR (50 µg/mL). Step corrections were applied to both the start of association and dissociation. Finally, the individual experiments were aligned to the start of association (x=y=0 for t=180 sec).

### 2.7 Isolation of peripheral blood mononuclear cells and T cells

Buffy coats were purchased from Sanquin (nr. NVT0465), and all donors gave informed consent (Sanquin Blood Supply, Groningen, the Netherlands). Human peripheral blood mononuclear cells (PBMCs) were isolated *via* density gradient centrifugation using lymphoprep (cat# 07851/07861, STEMCELL Technologies, Vancouver, Canada) and frozen until the day of the assay. T cells were isolated from fresh PBMCs using an autoMACS Pro Separator (Miltenyi Biotec, Bergisch Galdbach, Germany) and a Pan T Cell Isolation Kit (cat# 130-096-535, Miltenyi Biotec) following the manufacturer’s recommendations. After isolation, T cells were frozen until the day of the assay.

### 2.8 CD27xEGFR binding studies

Binding of CD27xEGFR to CD27 and EGFR was evaluated using cell lines A431, HT1080.CD27, and primary human T cells. In brief, 5x10^4^ cells were incubated with CD27xEGFR (0.01–10 µg/mL, 45 min at 4°C) in a 96 well plate (cat# 3799, Corning Inc.) washed 3 times with DPBS (cat# 14190144, Gibco, Thermo Fisher Scientific), and then incubated with anti-human-IgG-PE pAb (45 min at 4°C). Following 3 washes with DPBS, cells were evaluated by flow cytometry (Accuri C6 Plus Flow Cytometer, BD, Franklin Lakes, NJ, USA). Binding to primary human T cells was performed analogously, but in the presence of FcR blocking reagent (cat# 130-059-901, Miltenyi Biotec) in all incubation steps. To demonstrate EGFR-specific binding of CD27xEGFR, HT1080.CD27 cells were pre-incubated with a 10-fold molar excess of mAb 425 for 15 min at 4°C. To demonstrate CD27-specific binding of CD27xEGFR, HT1080.CD27 cells were pre-incubated with a 10-fold molar excess of CD27 mAb for 15 min at 4°C. Binding of CD27xEGFR to HT1080.CD27 was blocked by pre-incubation with a 10-fold molar excess of CD27 mAb and mAb 425. The mean fluorescent intensity (MFI) was normalized to the highest obtained MFI (which was set at 100%) with the 0 µg/mL CD27xEGFR condition being set at 0%. Doublet formation between A431 tumor cells and primary human T cells upon addition of CD27xEGFR was analyzed by pre-labeling A431 cancer cells with Vybrant DiD Cell-Labeling Solution (cat# V22887, Thermo Fisher Scientific) and primary human T cells with CellTrace Violet reagent (cat# C34557, Thermo Fisher Scientific) both according to manufacturer’s protocol). Cells were subsequently mixed at a 1:1 ratio (2x10^5^ cells) with or without the addition of 10 μg/mL CD27xEGFR for 45 min at 4°C. Doublet formation was analyzed by flow cytometry (CytoFLEX V5-B5-R3, Beckman Coulter Life Sciences, Indianapolis, IN, USA).

### 2.9 Validation of scFvCD3 T cell activation system

In a 96 well plate (cat# 167008, Thermo Fisher Scientific), 100 μL of media containing 1x10^4^ primary PBMCs were added to 100 μL of media with or without CD27xEGFR (10 µg/mL) containing MDA-MB-231^WT^ or MDA-MB-231^scFvCD3^ cells previously incubated overnight at Effector : Target (E:T) ratios of 1:1, 1:2 and 1:5. After a 24-hour incubation, images were taken at 5x magnification using an EVOS FLoid Imaging System (cat# 4471136, Thermo Fisher Scientific) to visualize T cell clustering. Furthermore, PBMC cells were collected, stained for CD3 and CD25 and the CD25 expression of CD3^+^ cells was measured using flow cytometry (CytoFLEX V5-B5-R3).

### 2.10 T cell proliferation assay

In a 96 well plate (cat# 167008, Thermo Fisher Scientific), 100 μL of media containing 4x10^4^ primary human T cells labeled with CellTrace Violet reagent with or without CD27xEGFR (10 µg/mL) were added to 100 μL of media containing 2x10^3^ ES-2^scFvCD3^, DLD-1^scFvCD3^ or FaDu^scFvCD3^ cells previously incubated overnight. Proliferation was measured using flow cytometry (CytoFLEX V5-B5-R3) on day 5 and quantified using FlowJo Software version 10.8.1.

### 2.11 T cell activation assay

In a 96 well plate (cat# 167008, Thermo Fisher Scientific), 100 μL of media containing primary human T cells with or without CD27xEGFR (10 µg/mL) were added to 100 μL of media containing 2x10^3^ ES-2^scFvCD3^, DLD-1^scFvCD3^ or FaDu^scFvCD3^ cells previously incubated overnight in the E:T ratios indicated (5:1, 10:1, 20:1). After 4 days, T cells were collected from the co-culture and stained for CD4, CD8, CD25, and Zombie NIR (cat# 423106, BioLegend) (to distinguish between alive and dead cells) and analyzed by flow cytometry (CytoFLEX V5-B5-R3). Supernatants were harvested and IFN-γ secretion was quantified using an IFN-γ ELISA kit (cat# 31673539, ImmunoTools, Friesoythe, Germany).

### 2.12 Cytotoxicity assay

In a 96 well plate (cat# 167008, Thermo Fisher Scientific), 100 μL of media containing primary human T cells with or without CD27xEGFR (10 µg/mL) were added to 100 μL of media containing 2x10^3^ ES-2^scFvCD3^, DLD-1^scFvCD3^, FaDu^scFvCD3^, OVCAR-3^scFvCD3^, or OVCAR-3^scFvCD3^EGFR^KO^ cells incubated overnight in the E:T ratios indicated (0:1, 1:1, 2:1, 5:1, 10:1, 20:1). Experiments were imaged for mCherry fluorescence for up to 7 days using the Incucyte S3 live-imaging system (Essen BioScience, Royston, UK) and analyzed using Incucyte S3 software v2021A. Four pictures of each well for each of three technical replicates were acquired and analyzed based on the Top-Hat segmentation method (Radius 50 µm, Threshold 0.0950, Edge Split On, Edge Sensitivity 5, Hole Fill 0 µm^2^, Adjusted Size 7 pixels, Filters: min Area 210 µm^2^, min Integrated Intensity 50). As a measure of cytotoxicity, cell survival was calculated as the mCherry area (µm²/image) from the sample at the indicated time point/mCherry area (µm²/image) from the cancer cells only control at the indicated time point. To evaluate direct EGFR-blocking anti-carcinoma activity of CD27xEGFR, 2x10^3^ mCherry expressing FaDu^scFvCD3^ cells were seeded in a 96 well plate (cat# 167008, Thermo Fisher Scientific) and treated with CD27xEGFR (10 µg/mL) or mAb425 (10 µg/mL) for 3 days.

### 2.13 Statistical analysis

Data are presented as mean + SD as stated in the figure legends. For the before-after plots, each pair of observations represents independent experiments with different T cell donors. Statistical significance was determined as indicated in the figure legends, with a p-value of < 0.05 considered statistically significant.

For the proportions of CD27^+^ cells, a two-sample test for equality of proportions with Bonferroni correction was applied for comparing the proportions of CD27 among different T cell types. The corresponding p-values are documented in **Supplementary Table 2**.

For CD27xEGFR binding, binding blockade, doublet formation, T cell proliferation, cytotoxicity, and IFN-γ secretion, experiments were performed with T cells from different donors on different days, and each experiment was treated as independent. The normality of flow cytometry data was assessed through visual inspection of flow cytometry histograms. For the proliferation and IFN-γ secretion data, normal distribution was assumed. For the cytotoxicity experiments, the Shapiro-Wilk test was employed to determine the normality of the data on three independent experiments consisting of three technical replicates.

For the relationship between EGFR or scFvCD3 expression and difference of cancer cell survival between CD27xEGFR and medium control, a simple linear regression was performed. Each data point in the analysis represents the mean of all independent experiments at the E:T 5:1, conducted for each cell line with different T cell donors.

## 3 Results

### 3.1 CD27 is a target for re-activation of tumor infiltrating cytotoxic and exhausted lymphocytes

**Figure 1:**
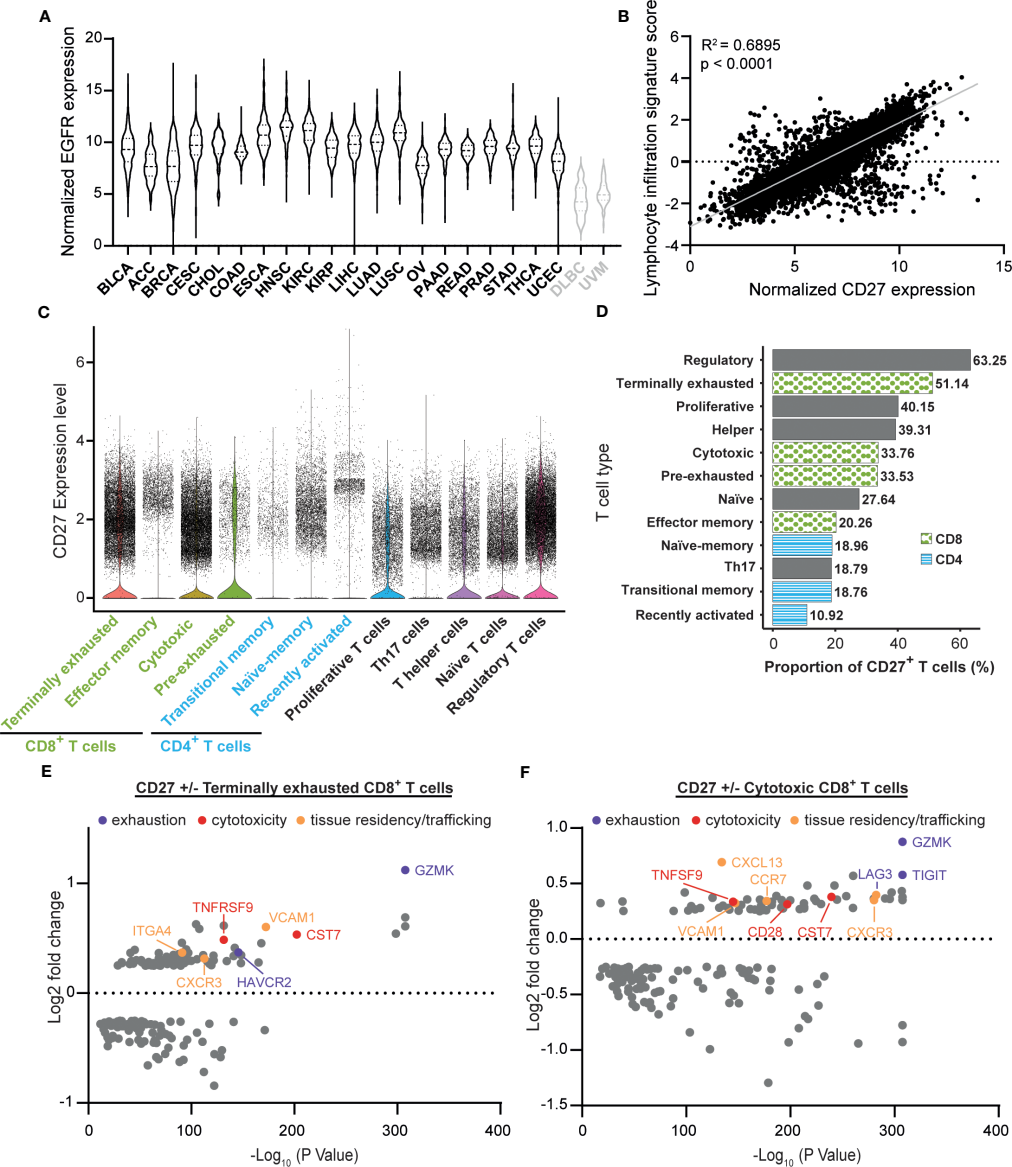
CD27 is a target for re-activation of tumor infiltrating cytotoxic and exhausted lymphocytes. **(A)** Normalized TCGA PAN CANCER epidermal growth factor receptor (EGFR) expression levels from epithelial cancers (black): BLCA (Bladder urothelial carcinoma), ACC (Adrenocortical carcinoma), BRCA (Breast invasive carcinoma), CESC (Cervical squamous cell carcinoma and endocervical adenocarcinoma), CHOL (Cholangiocarcinoma), COAD (Colon adenocarcinoma), ESCA (Esophageal carcinoma), HNSC (Head and Neck squamous cell carcinoma), KIRC (Kidney renal clear cell carcinoma), KIRP (Kidney renal papillary cell carcinoma), LIHC (Liver hepatocellular carcinoma), LUAD (Lung adenocarcinoma), LUSC (Lung squamous cell carcinoma), OV (Ovarian serous cystadenocarcinoma), PAAD (Pancreatic adenocarcinoma), READ (Rectum adenocarcinoma), PRAD (Prostate adenocarcinoma), STAD (Stomach adenocarcinoma), THCA (Thyroid carcinoma), UCEC (Uterine Corpus Endometrial Carcinoma), and non- epithelial cancers (gray): DLBC (Diffuse large B-cell lymphoma) and UVM (Uveal melanoma) were plotted in violin plots to visualize their relative EGFR expression. **(B)** Normalized CD27 expression levels from all 20 epithelial cancer types described in **(A)** were matched with lymphocytic infiltration signature scores *via* TCGA participant barcodes and plotted against each other. A linear regression was performed to visualize the correlation between CD27 expression and the lymphocytic infiltration signature score (R-squared = 0.6895, p < 0.0001). Statistical significance was determined using an F-test. **(C)** Single-cell tumor immune atlas RNA sequencing dataset based on 526,261 cells from 217 patients and 13 cancer types, revealing CD27 expression within different immune cell subtypes. **(D)** Proportion of CD27^+^ cells in each T cell type described in **(C)**, statistical comparisons are shown in **Supplementary Table 2**. **(E)** Volcano plots of the differential gene expression analysis in CD27^+^ vs CD27^-^ terminally exhausted and **(F)** cytotoxic CD8^+^ T cells calculated using the FindMarkers function from Seurat with MAST as the method of choice.

To delineate the potential applicability of EGFR-targeted activation of CD27 agonism, the TCGA PAN CANCER dataset was analyzed for concurrent CD27 and EGFR expression. Compared to non-epithelial cancers, lymphoid neoplasm diffuse large B-cell lymphoma (DLBC), and uveal melanoma (UVM), all 20 epithelial cancers expressed high levels of EGFR mRNA (**Figure 1A**, black for epithelial and gray for non-epithelial cancers). Furthermore, lymphocytic infiltrates across all 20 epithelial cancers expressed CD27 mRNA, demonstrating a clear correlation between lymphocyte infiltration score and CD27 (R^2^ = 0.6895) within the whole epithelial cancer set, confirming that the strategy of EGFR-mediated crosslinking of CD27 could be employed within epithelial cancers (**Figure 1B**, see **Supplementary Figure 2** for individual tumor types). In an established single-cell tumor immune atlas from a range of cancer types, CD27 expression was prominent in a subpopulation of regulatory T cells (T_regs_), terminally exhausted CD8^+^ T cells, as well as cytotoxic CD8^+^ T cells (**Figure 1C**). Notably, tumor-reactive CD8^+^ T cells - inclusive of cytotoxic, terminally exhausted, and pre-exhausted cells - present a higher proportion of CD27^+^ cells in comparison to all CD4^+^ T cells, Th17 cells, and naïve T cells (**Figure 1D**). Upon comparing CD27^+^ T cells proportions (**Figure 1D**, **Supplementary Table 2**), T_regs_ and terminally exhausted CD8^+^ T cells have proportions of 63.25% and 51.14% that are significantly higher than those observed in other T cell types. Notably, the proportions of CD27^+^ cells within cytotoxic and pre-exhausted CD8^+^ T cells are similar, with no significant difference observed between these two groups (**Supplementary Table 2**). Subsequent differential gene expression analysis in terminally exhausted CD8^+^ and cytotoxic CD8^+^ T cell subsets revealed that several exhaustion genes (GZMK, HAVCR2, TIGIT, and LAG3) and cytotoxicity genes, (TNFRSF9, CST7, and CD28) are significantly upregulated in the CD27^+^ fraction (**Figures 1E, F**). In addition, expression of genes associated with tissue residency, trafficking, adhesion, and migration (VCAM1, CXCR3, ITGA4, CXCL13, and CCR7) are also elevated (**Figures 1E, F**). Therefore, the concurrent expression of CD27 and EGFR in various epithelial cancers, along with the gene expression signatures related to exhaustion, cytotoxicity, and tissue residency of CD27-expressing T cells, underscores the potential of EGFR-targeted activation of CD27 agonism for enhancing the re-activation of tumor-infiltrated and tumor-reactive T cells in EGFR-expressing cancers.

### 3.2 Bispecific antibody CD27xEGFR binds selectively and simultaneously to EGFR and CD27

**Figure 2:**
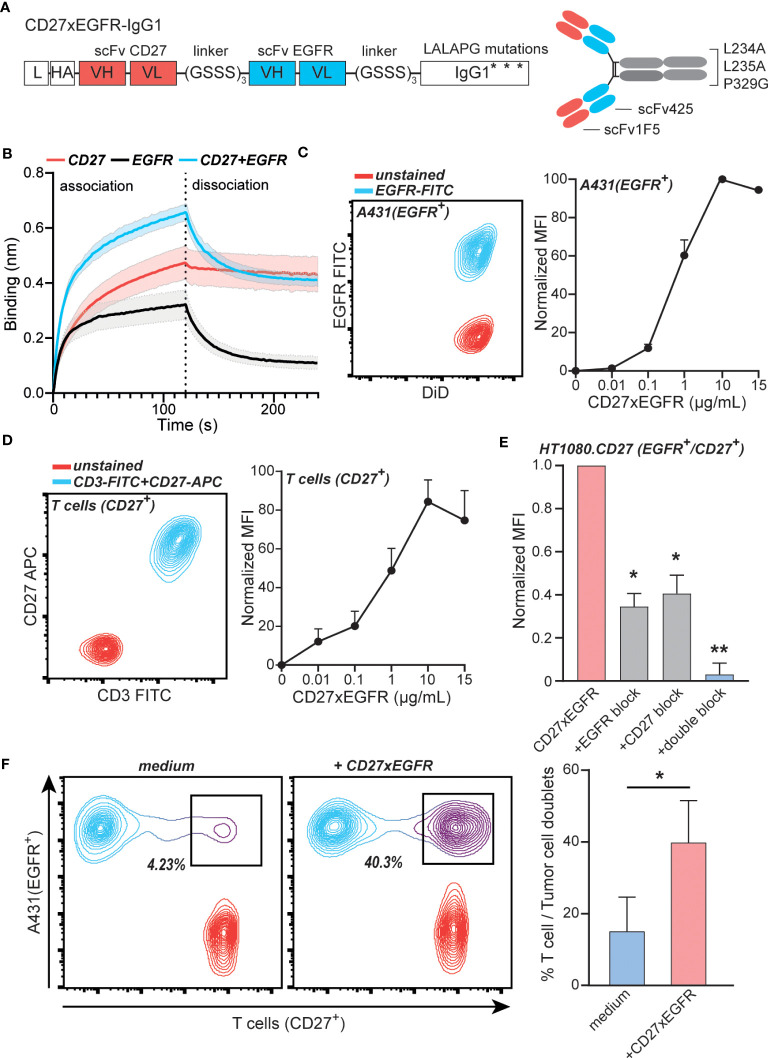
CD27xEGFR selectively binds EGFR and CD27 on tumor cells and T cells. **(A)** CD27xEGFR is designed in a scFv-scFv-IgG1 format with binding domains targeting CD27 (scFv1F5) and EGFR (scFv425) connected to an IgG1 tail containing LALAPG Fc mutations L234A, L235A, and P329G. **(B)** Association and dissociation of His-CD27 (500 nM) and/or His-epidermal-growth-factor-receptor (EGFR) (125 nM) against surface bound CD27xEGFR (8 µg/mL) as measured by biolayer interferometry (n = 3). **(C)** Flow cytometry plot displaying EGFR expression of stained (DiD) A431 cells (left). Dose-dependent binding (represented as normalized mean fluorescent intensity (MFI) to the highest MFI value) of CD27xEGFR on A431 tumor cells (n = 3) (right). **(D)** Flow cytometry plot displaying CD3 and CD27 expression on primary human T cells (left) Dose-dependent binding (represented as normalized MFI to the highest MFI value) of CD27xEGFR on primary human T cells (n = 5) (right). **(E)** Binding (represented as normalized MFI to the highest MFI value) of CD27xEGFR to HT1080 tumor cells ectopically expressing CD27 and its (partial) binding abrogation by pre-incubation of excess amounts of mAb 425 (EGFR block), an anti-CD27 mAb (CD27 block) or both (double block) (n = 3). Statistical significance was determined using one-way ANOVA test with Dunnett’s correction **(F)** Representative doublet formation between EGFR^+^ A431 tumor cells and CD27^+^ primary human T cells upon incubation with CD27xEGFR with the corresponding bar graph on the right (n = 3). Statistical analyses were done using a paired t-test. Data are presented as mean with shaded areas and error bars denoting standard deviation. “**” indicates (p < 0.01), “*” indicates (p < 0.05).

To exploit the above-described concurrent expression of EGFR and CD27 for targeted activation of CD27 signaling, the ICS-bsAb CD27xEGFR was constructed. CD27xEGFR consists of an N-terminal CD27 targeting antibody fragment (scFv1F5) fused *via* a (GGGGS)_3_ linker to the EGFR-targeting antibody fragment scFv425, with a silent human IgG1 containing LALAPG mutations (**Figure 2A**), which prevent Fc-FcR mediated antibody effector functions. The specific binding activity of CD27xEGFR to soluble CD27 and EGFR individually was confirmed using biolayer interferometry (**Figure 2B**). Moreover, the association rate (as defined by the gradient of the initial association curve) increased when both antigens were combined compared to a single antigen, supporting the proposed mechanism of action of dual binding (**Figure 2B**). Reversely, upon immobilization of CD27 or EGFR onto the biosensor, CD27xEGFR also specifically bound to both targets (**Supplementary Figure 1B**).

In a cell-based assay, CD27xEGFR dose-dependently bound to the EGFR^+^ epidermoid carcinoma cell line A431 (**Figure 2C**). Similarly, dose-dependent binding of CD27xEGFR was detected on CD27^+^ primary human T cells that expressed CD27 but not EGFR (**Figure 2D**, **Supplementary Figure 1C**). Further, CD27xEGFR also bound to the EGFR^+^ fibrosarcoma cell line HT1080, engineered to ectopically express CD27, with binding only partly inhibited by pre-incubation with mAb 425 (anti-EGFR mAb) or anti-CD27 mAb alone (**Figure 2E**). However, CD27xEGFR binding was abrogated after a combined pre-incubation with mAb 425 and anti-CD27 mAb (**Figure 2E**), demonstrating that CD27xEGFR binds to both antigens when they are present on the same cell surface. Moreover, CD27xEGFR induced the formation of doublets between EGFR^+^ and CD27^+^ target cells in a mixed culture of A431 and primary human T cells. The percentage of doublets increased significantly from ~15% in the medium control up to ~40% in the CD27xEGFR-treated condition (**Figure 2F**), demonstrating CD27xEGFR simultaneously interacted with EGFR and CD27 expressed on distinct cells. In conclusion, the designed bsAb CD27xEGFR exhibited selective and simultaneous binding to both targets (CD27 and EGFR).

### 3.3 CD27xEGFR enhances T cell proliferation and activation upon TCR stimulation

**Figure 3:**
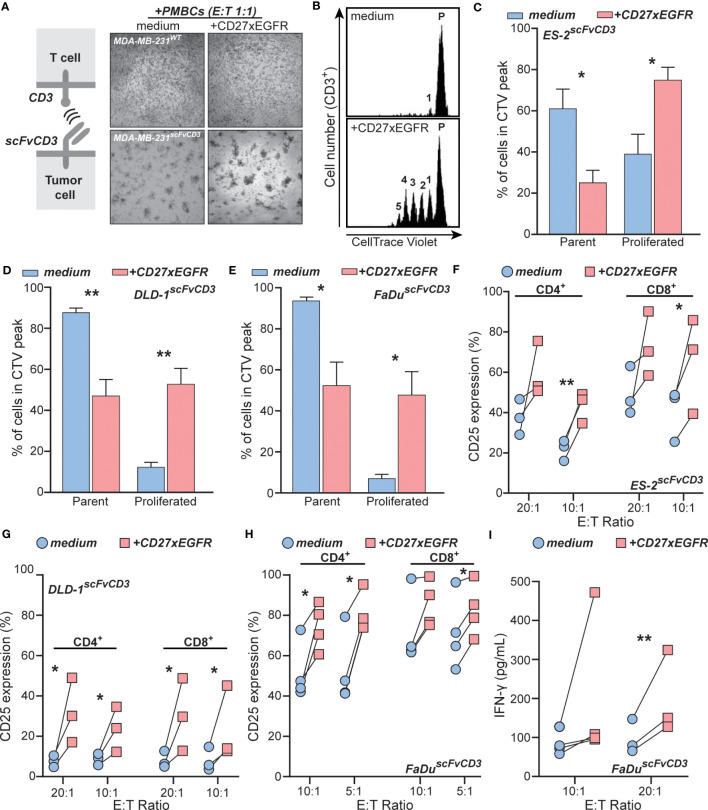
CD27xEGFR enhances T cell proliferation and activation upon TCR stimulation. **(A)** Schematic representation of the anti-CD3 (scFvCD3) T cell activation system (left). Microscopy images of MDA-MB-231^scFvCD3^ or MDA-MB-231^WT^ cells co-cultured with peripheral blood mononuclear cells (PBMCs) with or without the addition of CD27xEGFR (10 µg/mL) at an Effector : Target (E:T) ratio of 1:1 for 24 hours (right). **(B)** An exemplary co-culture and proliferation analysis of CellTrace Violet-labeled primary human T cells and ES-2^scFvCD3^ for 5 days with or without the addition of CD27xEGFR (10 µg/mL). Quantification analysis of proliferation peaks in co-culture experiments of primary human T cells with **(C)** ES-2^scFvCD3^ (n = 6), **(D)** DLD-1^scFvCD3^ (n = 3), and **(E)** FaDu^scFvCD3^ (n = 3) with (red bars) or without (blue bars) CD27xEGFR (10 µg/mL). Analysis of CD25 expression on CD4^+^ and CD8^+^ T cells after a 4-day co-culture experiment of **(F)** ES-2^scFvCD3^ (n = 3), **(G)** DLD-1^scFvCD3^ (n = 3), and **(H)** FaDu^scFvCD3^ (n = 4) with (red squares) or without (blue circles) the addition of CD27xEGFR (10 µg/mL) at the indicated E:T ratios. **(I)** ELISA analysis of co-culture supernatants for IFN-γ secretion by primary human T cells after a 4-day co-culture of FaDuscFvCD3 cells with (red squares) or without (blue circles) CD27xEGFR (10 µg/mL) at the indicated E:T ratios (n = 3 or 4). Significance was determined using paired t-tests. Data are presented as mean with error bars indicating standard deviation. “**” indicates (p < 0.01),”*” indicates (p < 0.05).

To evaluate the EGFR-restricted co-stimulation of T cells by CD27xEGFR, the carcinoma cell lines MDA-MB-231, ES-2, DLD-1, and FaDu were engineered to express a UchtV1 anti-CD3 antibody fragment (scFvCD3) on their surface, which enabled activation of TCR signaling in allogeneic T cells independent of MHC presentation (**Figure 3A**). The system was validated by treating a culture of MDA-MB-231^scFvCD3^ with peripheral blood mononuclear cells (PBMCs), which clearly activated T cells, as evidenced by cluster formation in the MDA-MB-231^scFvCD3^ co-culture (**Figure 3A**, bottom left). The addition of CD27xEGFR to this co-culture increased cluster formation further (**Figure 3A**, bottom right). Using flow cytometry, an increase in CD25 expression was observed in T cells within the PBMC population upon treatment with CD27xEGFR compared to the medium control (**Supplementary Figure 1D**). In contrast, no cluster formation was detected in the co-culture of MDA-MB-231^WT^ with PBMCs with or without CD27xEGFR treatment (**Figure 3A**, top left and right), validating that scFvCD3 can activate T cells MHC-independently. To specifically study the effects of CD27xEGFR on T cells, T cells were isolated from PBMCs in further studies. In line with the cluster formation, a prominent proliferation of T cells was detected in CD27xEGFR-treated mixed cultures with ES-2^scFvCD3^ cells, with up to 5 proliferation peaks detected (**Figure 3B**, bottom). In contrast, minimal proliferation was detected in the mixed cultures of ES-2^scFvCD3^ and T cells in the absence of CD27xEGFR (**Figure 3B**, top). Upon quantification, a significant increase in T cell proliferation was detected in CD27xEGFR treated ES-2^scFvCD3^ co-cultures, as evidenced by a significantly reduced percentage of cells in the parental peak and an increased percentage of cells in the proliferation peaks compared to medium control co-cultures (**Figure 3C**). A similar co-stimulatory activity of T cells by CD27xEGFR was detected in mixed cultures with DLD-1^scFvCD3^ and FaDu^scFvCD3^, with a significant increase in T cell proliferation in either culture upon CD27xEGFR treatment (**Figures 3D, E**, respectively). Consistent with this increase in proliferation, CD27xEGFR treatment of ES-2^scFvCD3^, DLD-1^scFvCD3^, and FaDu^scFvCD3^ co-cultures increased the expression of CD25 on T cells compared to medium control co-cultures (**Figures 3F–H**, respectively), both on CD4^+^ and CD8^+^ T cells, and at different E:T ratios. The largest increase was detected at an E:T ratio of 20:1, with a 20–25% increase. Finally, CD27xEGFR treatment increased pro-inflammatory cytokine IFN-γ secretion in FaDu^scFvCD3^ co-cultures at 10:1 and 20:1 E:T ratios compared to medium control co-cultures (**Figure 3I**). Taken together, this data provides evidence that CD27xEGFR can effectively co-stimulate T cells in co-cultures with a wide range of EGFR^+^ cell lines.

### 3.4 CD27xEGFR boosts T cell anti-tumor cytotoxic potential and has EGFR blocking anti-proliferative effects

**Figure 4:**
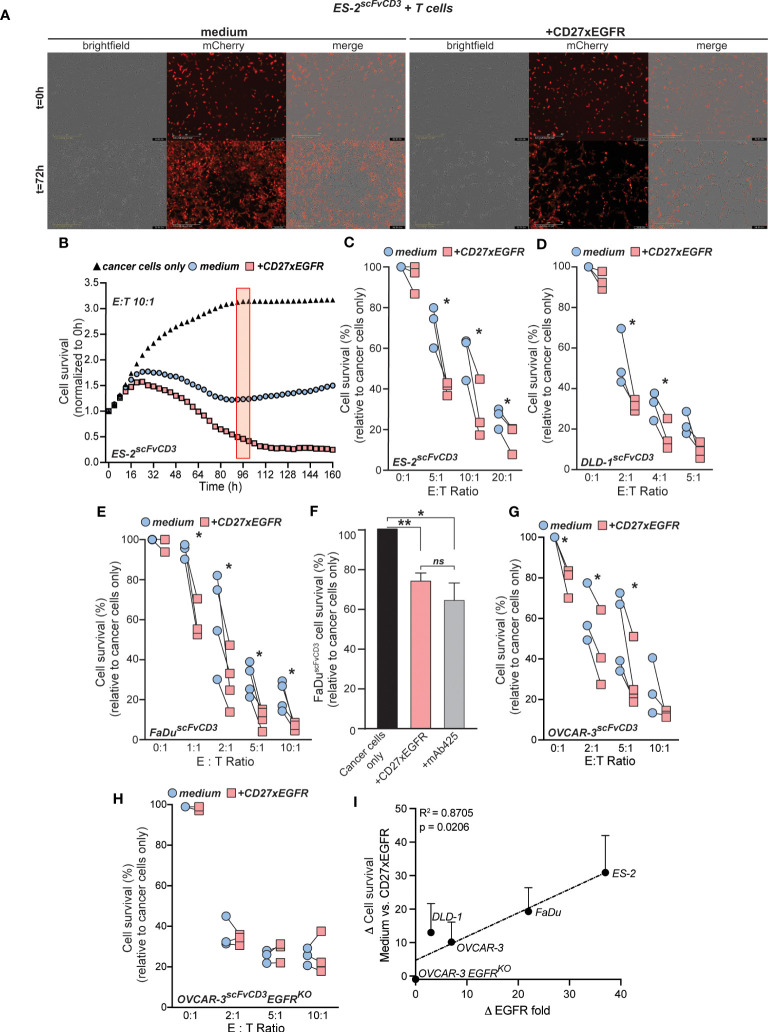
CD27xEGFR enhances T cell anti-tumor cytotoxic potential. **(A)** Exemplary brightfield microscopy images of a co-culture of mCherry-expressing ES-2^scFvCD3^ cells (overlayed in red) with primary human T cells for three days with or without the addition of CD27xEGFR (10 µg/mL) **(B)** ES-2^scFvCD3^ mCherry intensity (normalized to 0 h) over time using the IncuCyte S3 system (Essen BioScience) and analyzed at 96 h (red rectangle) using the IncuCyte 2021A software. The cancer cell survival (relative to cancer cells only, black triangles) of mCherry-expressing **(C)** ES-2^scFvCD3^ (n=3), **(D)** DLD-1^scFvCD3^ (n = 3), and **(E)** FaDu^scFvCD3^ (n = 3 or 4) after a 4 (ES-2^scFvCD3^), 4 (DLD-1^scFvCD3^), or 7 (FaDu^scFvCD3^) day co-culture with (red squares) or without (blue circles) the addition of CD27xEGFR (10 µg/mL) at the indicated Effector : Target (E:T) ratios. For each plot, pairs of data points represent independent experiments, each utilizing T cells from a unique donor. Statistical significance was determined using paired t-tests. **(F)** mCherry-expressing FaDu^scFvCD3^ cancer cell survival (relative to cancer cells only) in a 3-day co-culture treated with CD27xEGFR (10 µg/mL) or mAb 425 (10 µg/mL) (n = 4). Statistical significance was determined using a one-way ANOVA test with Tukey’s correction. **(G, H)** mCherry-expressing OVCAR-3^scFvCD3^ (n = 3 or 4) or OVCAR-3^scFvCD3^EGFR^KO^ (n = 3) cancer cell survival (% of cancer cells only) after a 3-day co-culture with primary human T cells with (red squares) or without (blue circles) the addition of CD27xEGFR (10 µg/mL) at the indicated E:T ratios. Statistical significance was determined using paired t-tests. **(I)** Linear regression depicting the positive correlation between higher EGFR expression levels and increased delta values between CD27xEGFR and medium control (R-squared = 0.8705, p = 0.0206). Each point on the graph represents an individual pair difference at the 5:1 E:T ratio. Statistical significance was determined using an F-test. Data are presented as mean with error bars indicating standard deviation. “**” indicates (p < 0.01), “*” indicates (p < 0.05), “n.s.” indicates non-significant differences.

In view of the clear co-stimulatory activity of CD27xEGFR, potential anti-tumor T cell activity induced by CD27xEGFR was evaluated next. As a measure of T cells cytotoxicity, cancer cell survival was determined by using the fluorescence of mCherry transduced cancer cells. After three days, treatment with CD27xEGFR strongly reduced the survival of mCherry-expressing ES-2^scFvCD3^ cells compared to medium control in co-culture experiments with primary human T cells (**Figure 4A**). To quantify this data, cell survival was measured over time. After approximately 24 hours of co-culture of T cells with mCherry-expressing ES-2^scFvCD3^ cells, T cell-mediated killing was observed, which continued to near complete eradication of cancer cells after 160 hours of treatment with CD27xEGFR (**Figure 4B**). In contrast, mCherry-expressing ES-2^scFvCD3^ started to grow back after approximately 96 hours when co-cultured with medium control and T cells (**Figure 4B**). Cell survival at this time point was normalized to that of cancer cells only and measured at different E:T ratios (0:1, 5:1, 10:1, and 20:1) (**Figure 4C**). An E:T ratio dependent reduction of cancer cell survival was detected, in which treatment with CD27xEGFR significantly reduced cancer cell numbers, with a maximum decrease of ~40% at an E:T ratio of 5:1 (**Figure 4C**). A similar reduction in cancer cell numbers in mCherry-expressing DLD-1^scFvCD3^ and mCherry-expressing FaDu^scFvCD3^ cells upon CD27xEGFR treatment was detected, with a maximum effect of ~20% and ~30% at a 2:1 E:T ratio for mCherry-expressing DLD-1^scFvCD3^ and mCherry-expressing FaDu^scFvCD3^ cells, respectively (**Figures 4D, E**). Of note, treatment of a monoculture of mCherry-expressing cancer cells (ES-2^scFvCD3^, DLD-1^scFvCD3^, and FaDu^scFvCD3^) with CD27xEGFR slightly reduced the survival of the cancer cells compared to the medium controls (**Figures 4C–E**, 0:1 E:T ratios, ~2-7%). This effect is most likely caused by the EGFR-growth inhibitory activity of CD27xEGFR, which proved to be reminiscent of EGFR blocking with mAb 425 (**Figure 4F**). Notably, in a co-culture of T cells with mCherry-expressing EGFR^+^ OVCAR-3^scFvCD3^, treatment with CD27xEGFR increased cancer cell killing by T cells (**Figure 4G**, ~25% reduction at an E:T ratio of 5:1) whereas with the corresponding EGFR^KO^ cells, treatment with CD27xEGFR had no effect on cancer cell survival when compared to medium control (**Figure 4H**). The EGFR and scFvCD3 expression levels varied between the cell lines (**Supplementary Figure 3**), with EGFR expression having a significant, positive correlation with the cytotoxicity of CD27xEGFR at an E:T ratio of 5:1 (**Figure 4I**). No correlation was identified for scFvCD3 levels (**Supplementary Figure 4**). Taken together, CD27xEGFR has anti-cancer activity both by co-stimulation of T cells at the sites of EGFR expression as well as by directly blocking EGFR on cancer cells.

## 4 Discussion

In this study, we identified that cytotoxic and exhausted CD8^+^ TILs with a tumor-reactive phenotype express the co-stimulatory receptor CD27 across various EGFR^+^ cancer subtypes. Our findings revealed that these CD27^+^CD8^+^ T cells display a cytotoxic, exhausted, and tumor-reactive profile, closely matching the reported dysfunctional profile of the tumor-reactive immune repertoire (54, 55).This observation underscores their potential as a target for immunotherapy and suggests that EGFR-targeted re-activation of CD27 co-stimulatory signaling T cells may have a broad applicability across a diverse range of carcinomas (54, 55). To therapeutically exploit this observation, we developed an Fc-silent ICS-bsAb that targets CD27 and EGFR. This bsAb was designed to be minimally active ‘en route’, while providing multivalent and tumor-localized crosslinking of CD27 when bound to EGFR^+^ cancer cells. Given the ubiquitous and abundant expression of EGFR in epithelial cells, it is important to account for potential toxicities that may arise from off-tumor targeting. Nevertheless, as the associated toxicities with targeting EGFR have remained within acceptable limits in the context of four clinically approved anti-EGFR mAbs (56), it is expected that side effects of co-stimulating CD27 through EGFR-targeting should not exceed these established thresholds. Supporting this concept, studies using a similar bispecific approach (CD28xEGFR), showed no independent stimulation of the immune system in the absence of TCR engagement with the MHC of cancer cells, as demonstrated in both cynomolgus monkeys and genetically engineered triple-humanized mice (57). In alignment with this data, our *in vitro* studies demonstrated CD27xEGFR simultaneously bound to both targets, enhanced T cell activation, increased T cell proliferation, and selectively potentiated anti-cancer T cell cytotoxicity. Additionally, based on the antagonistic properties of EGFR scFv, it is plausible to hypothesize that CD27xEGFR may have the potential to directly inhibit cell growth.

The tetravalent DVD-Ig antibody design of CD27xEGFR carries two EGFR and two CD27 antibody fragment domains (scFv 425 and scFv 1F5 targeting EGFR and CD27, respectively), facilitating bivalent binding to both targets. These specific scFvs were chosen based on their unique abilities to bind with and inhibit cell growth and trigger CD27 co-stimulation. Prior research by Murthy et al. established the efficacy of murine monoclonal antibody 425 in inhibiting the binding of EGF to its receptor, EGFR (58). The scFv variant of this antibody demonstrated moderate affinity to EGFR (200 nM < Kd < 400 nM) and exhibited growth inhibitory activity, as reported in previous studies (59–61). Similarly, the scFv 1F5, adapted from the agonistic mAb varlilumab (1F5), has the ability to block binding of soluble human CD70. It has exhibited significant preclinical activity, and is currently under evaluation in clinical trials (NCT03038672, and NCT04081688) (38). Therefore, although not formally investigated in this study, CD27xEGFR is expected to competitively inhibit ligand binding to the cognate receptors.

In the current study, CD27xEGFR was demonstrated to have high-affinity binding to EGFR on EGFR^+^ tumor cells and to CD27 on CD27^+^ T cells. Since the carcinoma and T cell binding domains are in-frame on either side, a potential concern could be that binding of one domain to its target cell would preclude binding to the second cell or domain. Although the two variable domains are indeed linked in tandem, the high domain flexibility of the DVD-Ig format was previously shown to allow for antigen binding of the inner domain with minimal steric hindrance (62). In line with this, CD27xEGFR demonstrated selective and simultaneous binding to EGFR and CD27, with clear doublet formation of carcinoma and T cells and inhibition of binding upon pre-incubation with excess amounts of mAb 425 and CD27 mAb. However, the possibility of T cell-to-T cell doublet formation as a result of CD27 binding on two different T cells has yet to be tested. Furthermore, CD27xEGFR had an increased association rate when exposed to both antigens, compared to a single antigen, as observed with biolayer interferometry, confirming the ability of CD27xEGFR to bind both targets simultaneously. This bsAb format is similar to recently described bsAbs that restrict immune checkpoint blockade of PD-1/PD-L1 or CD47 in an EGFR-restricted manner, leading to enhanced selectivity and efficacy of PD-L1 or CD47 blockade (63, 64). Based on the data presented here, CD27xEGFR may provide tumor-localized binding and crosslinking of CD27 on T cells for EGFR^+^ carcinomas.

CD27xEGFR mediated T cell proliferation and activation upon TCR stimulation occurred only in co-cultures with EGFR^+^ target cells, as evidenced by the increase in proliferating T cell peaks, the upregulation of CD25 expression in both CD4^+^ and CD8^+^ T cells, and increase in IFN-γ secretion. These results are consistent with previous studies showing that the CD27-targeting antibody Varlilumab upregulated T cell cytokine secretion (e.g., IFN-γ) and induced T cell proliferation at comparable levels in co-culture experiments (15, 33). Similarly, a tetravalent PD-L1 and CD27-targeting bsAb (CDX-527) induced IL-2 production upon TCR stimulation on a plate coated with OKT3 mAb and soluble PD-L1 (40). The functional activity of CDX-527 was further demonstrated using a CD27-NFκB reporter cell line, revealing enhanced activity compared to parental antibodies and further augmentation with the addition of recombinant soluble FcγR. However, CDX-527 relied on both PD-L1 expression and FcR interactions for CD27 crosslinking and activation, potentially unleashing strong on-target but off-tumor activity (65). CD27xEGFR’s FcR independence, relying on EGFR^+^ cancer cells to provide a CD27 cross-linking platform, could avoid these unwanted effects. Indeed, the combination of a T cell engager with the bsAb CD28xEGFR has successfully demonstrated the safe triggering of CD28 co-stimulatory signaling *via* EGFR crosslinking of co-stimulatory molecules, such as CD28 (57). Given that Varlilumab recently yielded synergistic anti-tumor activity in multiple tumor models when used in combination with PD-1/PD-L1 blockade (66), it would be worthwhile to further examine the activity of CD27xEGFR in combination with PD-1/PD-L1 blockade.

The antigen-dependent and tumor-selective cross-linking with CD27 was previously reported to functionally replace the FcγR dependent agonistic activity reported for several TNFRSF targeting antibodies (13, 67, 68). However, in a study that combined an EGFR-targeted bispecific T cell engager with several EpCAM-targeted TNFRSF bsAbs (41BB, OX40, TL1A, and CD27), the bsAb 41BBxEpCAM showed the highest activity (69). Therefore, it would be valuable to develop additional bsAbs with the same EGFR-selective tetravalent DVD-Ig antibody design but targeting different TNFRSF receptors, as these may provide even higher co-stimulatory activity than CD27.

CD27xEGFR-mediated cancer cell reduction was observed in different carcinoma cell line settings across a range of E:T ratios. The EGFR-dependent crosslinking of CD27 facilitated these effects, as the Fc domain of CD27xEGFR was designed with LALAPG mutations in order to create an effector silent IgG molecule to reduce off-target activity *via* FcγR-expressing cells. In line with the CD27 crosslinking requirements, previous studies with syngeneic mouse tumor models have shown that Varlilumab induces FcR-engagement-dependent tumor regression and facilitates long-term anti-tumor immunity (13). As silencing of the Fc domain in CD27xEGFR also excludes effector functions such as antibody-dependent cell-mediated cytotoxicity (ADCC) and complement-dependent cytotoxicity effects, a side-by-side comparison of Fc-silent CD27xEGFR to Fc-functional CD27xEGFR and their parental antibodies in co-culture experiments with PBMC populations should be conducted, next to experiments with isolated T cells. These molecules should also be evaluated as mouse surrogate molecules or in transgenic mice expressing human CD27 and EGFR, to further characterize the functional characteristics and safety profile of CD27xEGFR.

CD27xEGFR also had anti-proliferative effects, likely induced by blocking EGFR-mediated signaling, which was the strongest in 3-day treatments and comparable to the effects induced by mAb 425. This is consistent with an earlier report where mAb 425 and bsAb PD-L1xEGFR were compared for their ability to inhibit EGFR-mediated cancer cell proliferation (63). The extent of EGFR inhibitory effects varied among each of the carcinoma cell lines, which is in line with the varying levels of sensitivity to EGFR-inhibition reported for different tumor types (as reviewed in (56)). Furthermore, the activity of CD27xEGFR directly correlated with the EGFR expression in each cell line. This correlation could be attributed to enhanced growth inhibitory effects induced by EGFR blockade, or to heightened CD27 co-stimulation facilitated by greater CD27xEGFR binding. Importantly, blocking EGFR signaling can induce remodeling of the tumor microenvironment (TME) towards an immunoresponsive phenotype in non-small cell lung cancer (NSCLC) and inflammatory breast cancer (70–72). Thus, the potential antiangiogenic activity of EGFR-restricted CD27 co-stimulation warrants further investigation.

In addition to CD27 being expressed in cytotoxic and exhausted TILs, CD27 mRNA expression was also detected in tumor infiltrating regulatory T cells (T_regs_), suggesting possible unwanted co-stimulatory effects on T_regs_ by CD27xEGFR. In this respect, the development of T_regs_ and increased T_regs_ activity in the TME are linked to CD27 agonism by CD70^+^ tumor cells (73, 74). Upon prolonged T_reg_ stimulation, however, CD27 expression is downregulated and CD70 upregulated, leading to subsequent CD70-mediated T cell co-stimulation (75). Notably, in NSCLC tumors that develop EGFR-TKI refractory disease, CD70 is upregulated by refractory cancer cells (76). In tumor-bearing mice, this CD70 interacts constitutively with CD27^+^ T_regs_ during tumor development, thereby promoting T_reg_ expansion and preventing cytotoxic T cell responses (77, 78). Therefore, it will be important to study the specific effects of CD27 co-stimulation on T_regs_ in the context of restricted co-stimulation to a tumor antigen such as EGFR in relevant murine models and in combination with T_reg_ depleting strategies, including sorafenib treatment (79).

In clinical studies, the active Fc domain of Varlilumab induced ADCC-mediated CD27^+^ T_reg_ depletion, while providing co-stimulation to effector T cells in both hematological and solid tumors (21, 22). Indeed, in some patients, Varlilumab even triggered the development of *de novo* CD8^+^ anti-tumor responses (22). Hence, as CD27xEGFR has an inactive Fc domain, the effector function of T_reg_ depletion and its subsequent effects, such as possible *de novo* CD8^+^ responses, would be expected to be absent. However, a study inducing transient and deliberate CD27 agonism in CD27^+^ T_regs_ through dendritic cells demonstrated that T_regs_ partially lost their suppressive function and converted into CD4^+^ Th1 cells (80). Furthermore, CD27 co-stimulation is critical for the protection of CD8^+^ T cells against subsequent T_reg_ suppression and is necessary for the priming of new T cells (25, 80). Therefore, CD27 agonism is anticipated to be a beneficial intervention, even in malignancies with T_regs_. Moreover, CD27 agonism also enhances NK cell activation and proliferation, suggesting that these two additional anti-tumor mechanisms could also be explored in the context of CD27xEGFR treatment in follow-up studies (81, 82).

In conclusion, CD27xEGFR is a novel DVD-Ig bsAb targeting CD27 and EGFR, that has the potential to re-activate T cell immunity in EGFR^+^ carcinomas through its interaction with tumor-reactive and exhausted CD27^+^CD8^+^ TILs. Moreover, the Fc-silent format of CD27xEGFR enables tumor-localized binding and crosslinking of CD27 only at EGFR^+^ tumor sites, potentially enhancing its specificity and safety profile. These unique features of CD27xEGFR offer a compelling rationale for its further exploration in preclinical and clinical settings as a promising immunotherapeutic agent for EGFR^+^ tumors.

## Data availability statement

Publicly available datasets were analyzed in this study. This data can be found here: https://www.ncbi.nlm.nih.gov/geo/query/acc.cgi?acc=GSE158803, GSE158803.

## Ethics statement

All blood donors gave informed consent (nr. NVT0465, Sanquin Blood Supply, Groningen, the Netherlands).

## Author contributions

VM, LN, MV, VW, GH, MdB, and EB contributed to the conception of the study. VM, LN, MV, VW, and EB contributed to investigation. VM, HL, and VB provided bioinformatic analysis. VM, LN, MV, HL, and EB contributed to data curation and formal analysis of the study.VM, LN, MV, and EB wrote the manuscript and were involved in manuscript revision. All authors contributed to the article and approved the submitted version.
