# Supplementary material for: EGFR-selective activation of CD27 co-stimulatory signaling by a bispecific antibody enhances anti-tumor activity of T cells
Source: Front Immunol. 2023 Jul 20;14:1191866. doi: 10.3389/fimmu.2023.1191866 (PMC10399592; doi:10.3389/fimmu.2023.1191866)
Supplement: Supplementary file 1 [file DataSheet_1.pdf]

Supplementary Material

EGFR-selective activation of CD27 co-stimulatory signaling by a bispecific antibody enhances anti-tumor activity of T cells

Vinicio Melo[1], Levi Collin Nelemans[1], Martijn Vlaming[1], Harm Jan Lourens[1], Valerie Wiersma[1], Vrouyr Bilemjian[1], Gerwin Huls[1], Marco de Bruyn[2], Edwin Bremer*[1]

[1]Department of Hematology, University Medical Center Groningen, University of Groningen, Groningen, the Netherlands

[2]Department of Obstetrics & Gynecology, University Medical Center Groningen, University of Groningen, Groningen, the Netherlands

*** Correspondence:**

Prof. Dr. E Bremer (e.bremer@umcg.nl), Department of Hematology, University Medical Center Groningen, University of Groningen, Groningen, the Netherlands

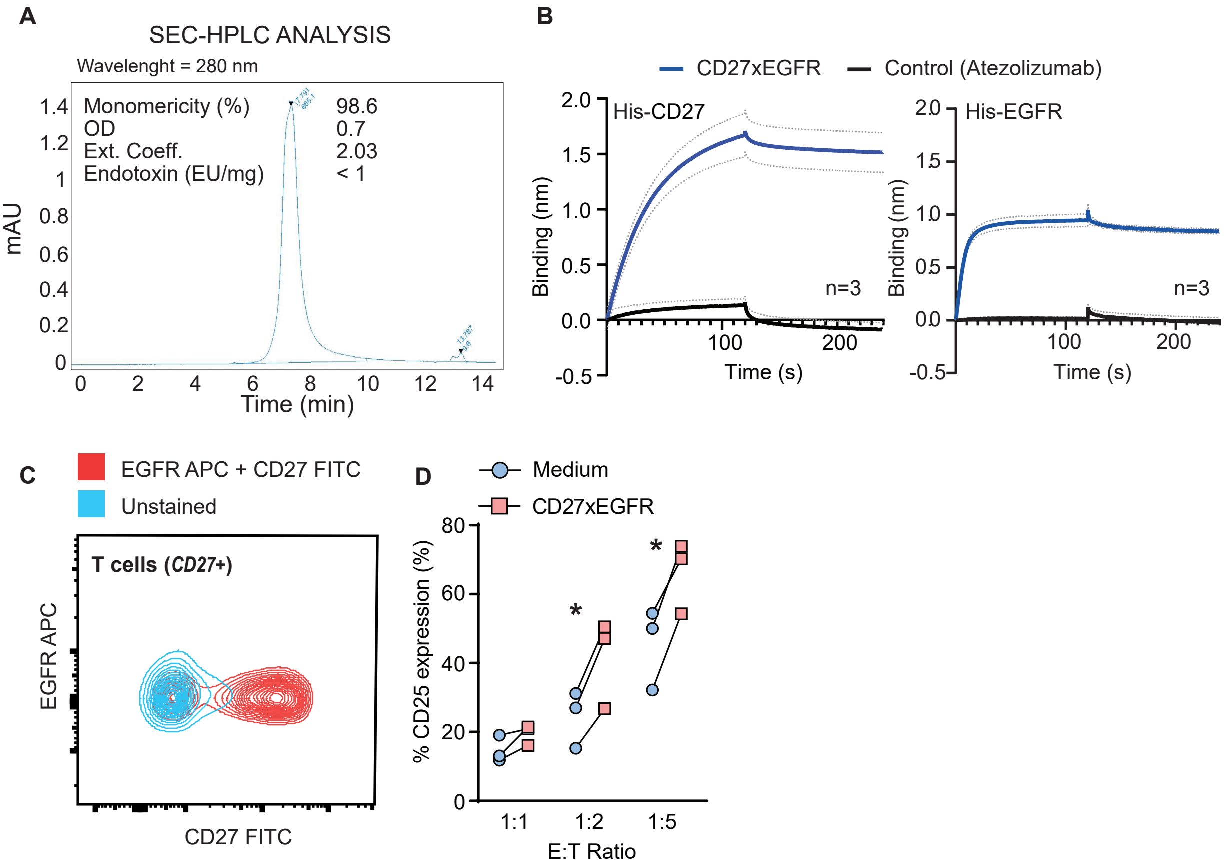

Supplementary Figure 1: Characterization of CD27xEGFR. (A) SEC-HPLC analysis of CD27xEGFR revealed high purity levels of 98,6% with only limited amounts of degradation product detected. Endotoxin levels were found to be < 1 EU / mg. **(B)** Association and dissociation of CD27xEGFR (50 µg/mL) and Atezolizumab (50 µg/mL) against surface-bound His-CD27 (500 nM) or His-EGFR (500 nM) as measured by biolayer interferometry (n = 3). Dotted lines indicate standard deviation. **(C)** Flow cytometry contour plot displaying EGFR and CD27 expression on primary human T cells. **(D)** Analysis of CD25 expression on $CD3^+$ T cells after a 24-hour co-culture experiment of MDA-MB-231scFvCD3 with (red squares) or without (blue circles) the addition of CD27xEGFR (10 µg/ml) at the indicated Effector:Target (E:T) ratios. Statistical significance was determined using a paired t-test. "*" indicates ($p<0.05$).

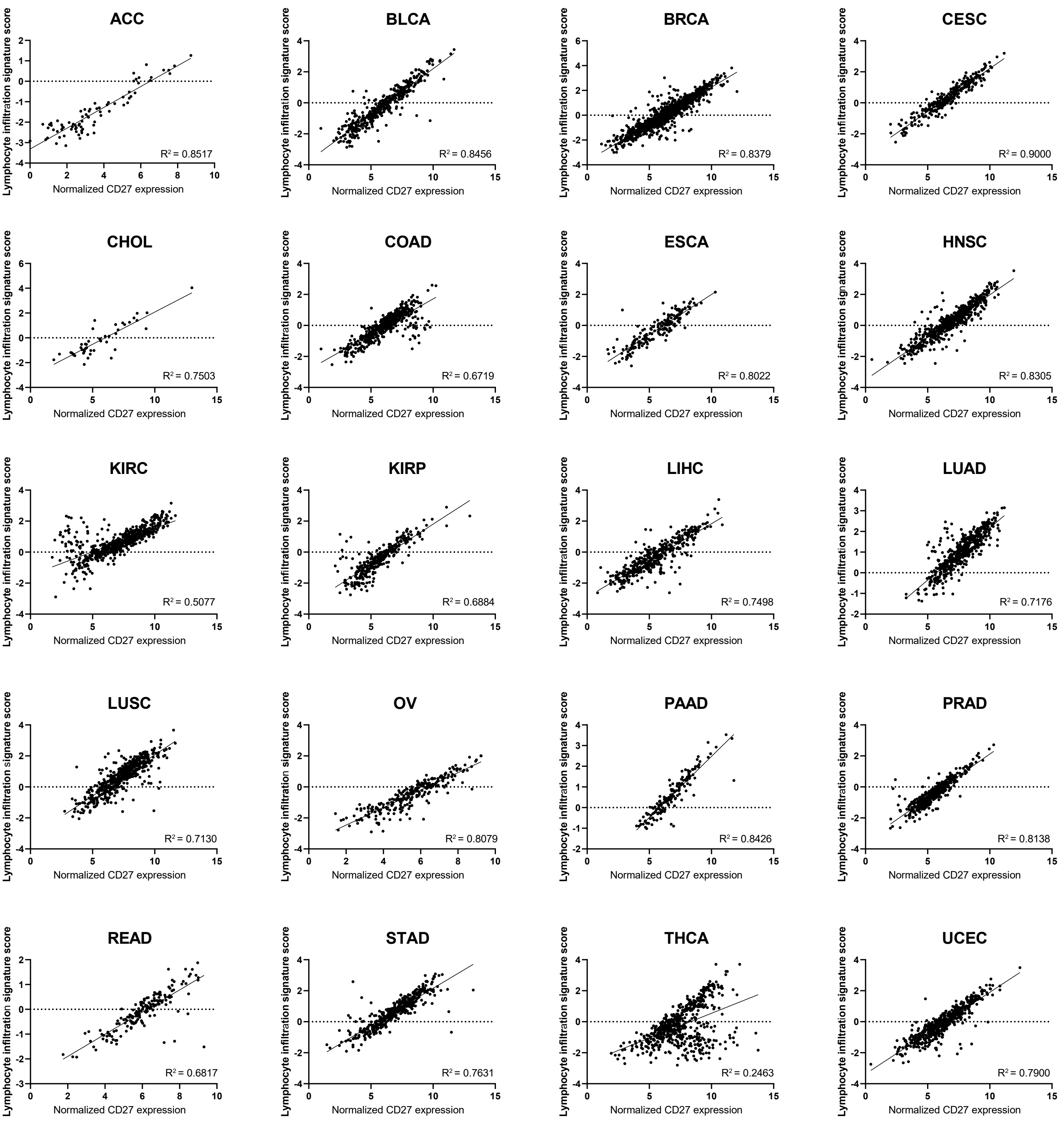

Supplementary Figure 2: Lymphocytic infiltrate correlates with CD27 expression in various solid tumor types

(A) Normalized TCGA PAN CANCER CD27 expression levels from ACC (Adrenocortical carcinoma), BLCA (Bladder urothelial carcinoma), BRCA (Breast invasive carcinoma), CESC (Cervical squamous cell carcinoma and endocervical adenocarcinoma), CHOL (Cholangiocarcinoma), COAD (Colon adenocarcinoma), ESCA (Esophageal carcinoma), HNSC (Head and Neck squamous cell carcinoma), KIRC (Kidney renal clear cell carcinoma), KIRP (Kidney renal papillary cell carcinoma), LIHC (Liver hepatocellular carcinoma), LUAD (Lung adenocarcinoma), LUSC (Lung squamous cell carcinoma), OV (Ovarian serous cystadenocarcinoma), PAAD (Pancreatic adenocarcinoma), PRAD (Prostate adenocarcinoma), READ (Rectum adenocarcinoma), STAD (Stomach adenocarcinoma), THCA (Thyroid carcinoma), and UCEC (Uterine Corpus Endometrial Carcinoma) matched with lymphocytic infiltration signature scores via TCGA Participant Barcodes and plotted against each other. A linear regression was performed to visualize the correlation between CD27 expression and lymphocytic infiltration signature scores across different tumor types.

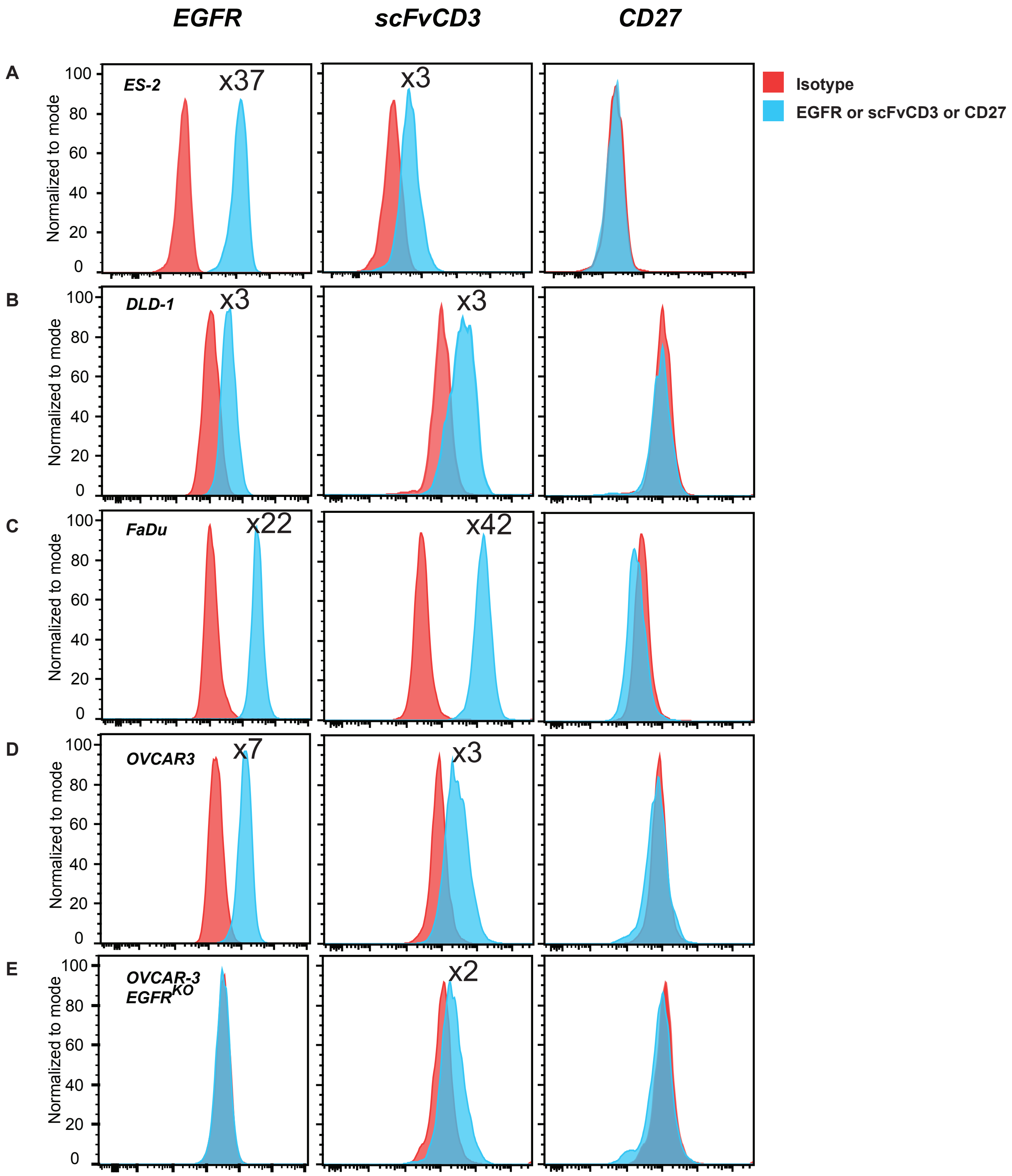

Supplementary Figure 3: Characterization of the fold change for EGFR, scFvCD3 and CD27 of the cancer cell lines used in the co-culture assays. (A) ES-2 **(B)** DLD-1 **(C)** FaDu **(D)** OVCAR-3 **(E)** OVCAR-3 EGFR.KO. "x" denotes the fold change over isotype control.

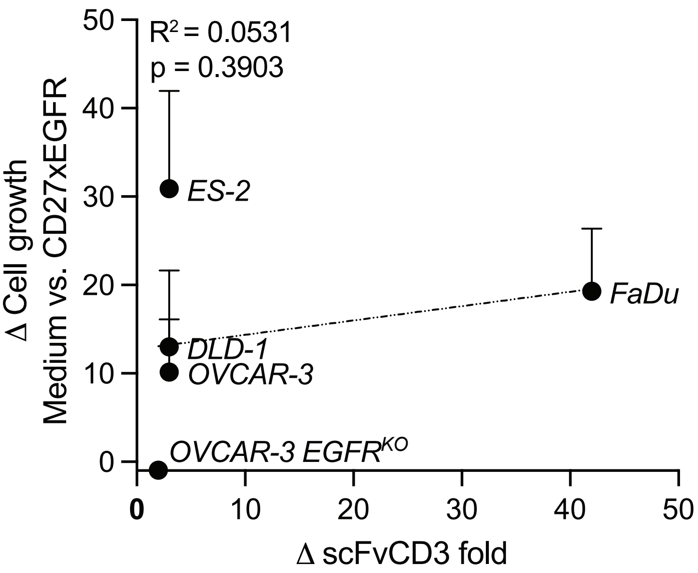

Supplementary Figure 4: Scatter plot depicting the correlation between scFvCD3 expression levels and delta values between CD27xEGFR and medium control. Each point on the graph represents an individual pair difference point from the study, plotted according to the scFvCD3 expression level of the corresponding cell line and the delta value between CD27xEGFR and the medium control. For ES-2, FaDu, DLD-1, OVAR-3, and OVCAR-3 $EGFR^{KO}$ (n = 3), and for FaDu (n = 4), with each data point in the scatter plot representing an independent co-culture experiment performed at a 5:1 Effector:Target (E:T) ratio, each using PBMCs from a different donor. (R-squared = 0.05315) (p = 0.3903).

T cell subset	Markers
Regulatory T cells	FOXP3, TNFRSF18, IL2RA, TIGIT, CTLA4, IKZF2
Terminally exhausted CD8 T cells	CXCL13, LAG3, GZMB, CCL5, NKG7, IFNG, GZMA, HAVCR2, GNLY, PDCD1, TIGIT, TNFRSF9, ENTPD1, CTLA4, PRF1, TOX, GZMH, GZMK
Proliferative T cells	STMN1, MKI67, CDK1
T helper cells	CXCL13, TNFRSF4, TNFRSF18, BATF, TIGIT, SOX4, TNFRSF25, CTLA4, RORA, XCL1, TNFSF8, PPIA, STAT5A, TOX, PDCD1
Cytotoxic CD8 T cells	CCL5, GZMA, GZMK, NKG7, GNLY, GZMH, GZMM, PRF1, CXCR3, GZMB, CCL4, IFNG
Pre-exhausted CD8 T cells	ISG15, IFI44L, IFI6, IFIT3, IFIT1, IFI44, IFI35, IRF7, IFIT2, LAG3, IFITM1, IFI16, IFI27, IFNG, GZMB, GZMK, PRF1, HAVCR2, IFIH1, GZMA, IRF9, CXCL13, GZMH, IFIT5, PDCD1
Naive T cells	IL7R, TCF7, CCR7, LEF1, SEL
Effector memory CD8 T cells	GZMM, IFITM1, GZMK, IFNG, CCL5
Naive-memory CD4 T cells	IL7R, TCF7, CCL5, IFITM1
Th17 cells	IL17A, IL17F, BATF, IL2RA, DUSP4, IL21R, CTLA4, IRF4, CCL20, IL26, BATF3, IL4R, STAT3, RORA
Transitional memory CD4 T cells	CXCL13, TNFRSF4, TIGIT, IL6ST, PASK, KLRB1, CD40LG, TOX, LEF1, ICOS, CD28, TOX2, CCR7, CD247, RORA, PDCD1
Recently activated CD4 T cells	CCL4, IFITM1, CD69, PRF1, BCL3, IL7R, IFITM3, TCF7, CD81, CXCR4, GZMK, GZMM, IFITM2

Supplementary Table 1: Key Markers for Identifying T Cell Subsets, Adapted from Nieto et al. (2020)

Cell type 1	Cell type 2	p value indicator
Regulatory T cells	Cytotoxic CD8 T cells	****
Regulatory T cells	Effector memory CD8 T cells	****
Regulatory T cells	Naive T cells	****
Regulatory T cells	Naive-memory CD4 T cells	****
Regulatory T cells	Pre-exhausted CD8 T cells	****
Regulatory T cells	Proliferative T cells	****
Regulatory T cells	Recently activated CD4 T cells	****
Regulatory T cells	T helper cells	****
Regulatory T cells	Terminally exhausted CD8 T cells	****
Regulatory T cells	Th17 cells	****
Regulatory T cells	Transitional memory CD4 T cells	****
Terminally exhausted CD8 T cells	Cytotoxic CD8 T cells	****
Terminally exhausted CD8 T cells	Effector memory CD8 T cells	****
Terminally exhausted CD8 T cells	Naive T cells	****
Terminally exhausted CD8 T cells	Naive-memory CD4 T cells	****
Terminally exhausted CD8 T cells	Pre-exhausted CD8 T cells	****
Terminally exhausted CD8 T cells	Proliferative T cells	****
Terminally exhausted CD8 T cells	Recently activated CD4 T cells	****
Terminally exhausted CD8 T cells	T helper cells	****
Terminally exhausted CD8 T cells	Th17 cells	****
Terminally exhausted CD8 T cells	Transitional memory CD4 T cells	****
Proliferative T cells	Cytotoxic CD8 T cells	****
Proliferative T cells	Effector memory CD8 T cells	****
Proliferative T cells	Naive T cells	****
Proliferative T cells	Naive-memory CD4 T cells	****
Proliferative T cells	Pre-exhausted CD8 T cells	****
Proliferative T cells	Recently activated CD4 T cells	****
Proliferative T cells	T helper cells	n.s.
Proliferative T cells	Th17 cells	****
Proliferative T cells	Transitional memory CD4 T cells	****
T helper cells	Cytotoxic CD8 T cells	****
T helper cells	Effector memory CD8 T cells	****
T helper cells	Naive T cells	****
T helper cells	Naive-memory CD4 T cells	****
T helper cells	Pre-exhausted CD8 T cells	****
T helper cells	Recently activated CD4 T cells	****
T helper cells	Th17 cells	****
T helper cells	Transitional memory CD4 T cells	****
Cytotoxic CD8 T cells	Effector memory CD8 T cells	****
Cytotoxic CD8 T cells	Naive T cells	****
Cytotoxic CD8 T cells	Naive-memory CD4 T cells	****
Cytotoxic CD8 T cells	Pre-exhausted CD8 T cells	n.s.
Cytotoxic CD8 T cells	Recently activated CD4 T cells	****
Cytotoxic CD8 T cells	Th17 cells	****
Cytotoxic CD8 T cells	Transitional memory CD4 T cells	****
Pre-exhausted CD8 T cells	Effector memory CD8 T cells	****
Pre-exhausted CD8 T cells	Naive T cells	****
Pre-exhausted CD8 T cells	Naive-memory CD4 T cells	****
Pre-exhausted CD8 T cells	Recently activated CD4 T cells	****
Pre-exhausted CD8 T cells	Th17 cells	****
Pre-exhausted CD8 T cells	Transitional memory CD4 T cells	****
Naive T cells	Effector memory CD8 T cells	****
Naive T cells	Naive-memory CD4 T cells	****
Naive T cells	Recently activated CD4 T cells	****
Naive T cells	Th17 cells	****
Naive T cells	Transitional memory CD4 T cells	****
Effector memory CD8 T cells	Naive-memory CD4 T cells	n.s.
Effector memory CD8 T cells	Recently activated CD4 T cells	****
Effector memory CD8 T cells	Th17 cells	*
Effector memory CD8 T cells	Transitional memory CD4 T cells	n.s.
Naive-memory CD4 T cells	Recently activated CD4 T cells	****
Naive-memory CD4 T cells	Th17 cells	n.s.
Naive-memory CD4 T cells	Transitional memory CD4 T cells	n.s.
Th17 cells	Recently activated CD4 T cells	****
Th17 cells	Transitional memory CD4 T cells	n.s.
Transitional memory CD4 T cells	Recently activated CD4 T cells	****

Supplementary Table 2: Adjusted p values from pairwise comparisons of CD27 proportions between T cell types. Results of a two-sample test for equality of proportions, used to compare the proportions of CD27 expression among different T cell types. P-values are adjusted to account for multiple comparisons using the Bonferroni correction. Each row represents a pairwise comparison between two distinct T cell types. "****" indicates ($p < 0.001$), "*" indicates marginal differences ($0.05 \leq p < 0.1$), n.s. indicates non-significant differences ($p \geq 0.1$).
